# Early occupational therapy use of a 3D printer introduced with minimal formal support: a single-site pre–post observational study

**DOI:** 10.3389/fresc.2026.1859684

**Published:** 2026-09-22

**Authors:** Yusuke Harada, Yuki Sawada, Hiroshi Momma, Rie Takeshima, Jun Suzurikawa

**Affiliations:** 1 Department of Rehabilitation, Faculty of Health Sciences, Kyorin University, Mitaka, Japan; 2 Department of Occupational Therapy, Faculty of Medical Sciences, Teikyo University of Science, Uenohara, Japan; 3 Department of Assistive Technology, Research Institute, National Rehabilitation Center for Persons with Disabilities, Tokorozawa, Japan

**Keywords:** 3D printing, early clinical use, early implementation phase, implementation research, occupational therapy

## Abstract

**Introduction:**

Three-dimensional (3D) printers can produce customized assistive devices in occupational therapy; however, occupational therapists' perceptions and early usage following clinical introduction remain poorly understood. This study examined perceptions, perceived barriers and enabling conditions, and early usage patterns among occupational therapists following 3D printer introduction in a rehabilitation hospital with minimal formal implementation support.

**Methods:**

A 3D printer was installed in the occupational therapy department, allowing trial printing of existing design data after brief basic instruction was provided to one representative therapist; no structured staff training, CAD instruction, formal usage recommendations, workflow integration, or dedicated institutional support was provided. A self-administered study-specific, non-validated questionnaire assessed perceptions and selected implementation-related conditions before and six months after installation. Paired data from 43 participants (follow-up rate: 79.6%) were analyzed using the Wilcoxon signed-rank and McNemar tests.

**Results:**

After installation, selection of the barrier “there is no 3D printer in the workplace” significantly decreased (adjusted *p* < 0.001), whereas selection of institutional and operational constraints, including insufficient time (adjusted *p* < 0.001) and lack of medical reimbursement (adjusted *p* = 0.044), increased. Participants showed less agreement that using a 3D printer was difficult (adjusted *p* = 0.047), while agreement that 3D printing was useful in occupational therapy practice also decreased (adjusted *p* = 0.044). Although statistically significant, these changes were small in magnitude. No statistically significant change was observed in future intention to use 3D printing (adjusted *p* = 0.063). Available printer records did not permit quantitative assessment of individual use; use remained limited to exploratory printing of existing designs.

**Discussion:**

The introduction of a printer with minimal formal support was not accompanied by clear progression toward routine clinical use during the six-month observation period. These exploratory findings support further evaluation of structured education, technical assistance, workflow integration, and institutional arrangements, but do not establish their effectiveness or the causal effect of printer availability.

## Introduction

In occupational therapy, assistive devices and welfare equipment play central roles in promoting independence in activities of daily living. However, because patients' conditions and living environments vary widely, commercially available products may not fully address individual needs. Therefore, occupational therapists have traditionally modified or fabricated assistive devices tailored to individual needs. Nevertheless, the implementation of such practices in clinical settings can be challenging due to constraints such as limited resources, time, and required expertise (1).

Recently, three-dimensional (3D) printing technology has been explored as a potential solution to these challenges. In the medical field, 3D printing has been applied to customized prosthetics and implants, patient-specific anatomical models, and pharmaceutical development (2). Within occupational therapy, it has demonstrated usefulness for rapid and flexible production of assistive devices and orthoses, offering reproducibility and precision advantages over traditional handcrafted methods (3, 4). In clinical practice, advanced applications include the production of individually fitted assistive devices, such as custom orthoses, using a combination of 3D scanning and printing (5).

Educational initiatives have been developed to enable occupational therapists to acquire 3D printing skills. Harada et al. (6) reported that a short-term (90-min session, 1 week apart) intensive training program improved occupational therapy students' understanding of and motivation to use 3D printing technology. Moreover, educational programs targeting rehabilitation students and professionals have demonstrated improvements in self-efficacy and attitudes toward 3D printing, suggesting a potential reduction in psychological barriers to its use (7–9).

Despite these developments, the clinical use of 3D printers in Japan remains limited. A nationwide survey reported that only 4.4% of 3,469 occupational therapists had experience using 3D printers (10); the main reported barriers were the absence of accessible equipment in the workplace and limited operational knowledge. Decision tree analysis indicated that, compared with individual characteristics, workplace availability and accessibility of 3D printers were more strongly associated with previous use experience (10), highlighting the importance of environmental factors in enabling initial exposure and use.

Therefore, physical access may be an important enabling condition for the clinical use of 3D printers, alongside education and individual motivation. However, equipment availability does not represent the full implementation environment, which may also include education, technical support, workflow integration, and institutional arrangements.

Implementation and technology-acceptance literature has identified concepts relevant to the introduction of new technologies, including acceptability, feasibility, appropriateness, adoption, perceived usefulness, and ease of use (11–13). Validated measures have also been developed to assess implementation outcomes such as acceptability, appropriateness, and feasibility systematically (14). However, no single implementation or technology-acceptance framework prospectively guided the study design, questionnaire development, outcome selection, or statistical analysis in the present study. These concepts were used only as broad background for interpreting occupational therapists' perceptions and early use, and the study was therefore treated as an exploratory descriptive investigation rather than a framework-based implementation study.

Although workplace availability may facilitate initial engagement with 3D printing, few studies have empirically examined changes in usage patterns or perceptions following clinical introduction with limited formal support. In the present study, the “early implementation phase” was operationally defined as the six-month period following installation during which the printer was physically accessible, one representative occupational therapist received brief basic operational instruction, pre-existing design files were provided, and information was shared informally among staff, but no structured training, CAD instruction, formal usage recommendations, workflow integration, dedicated personnel, or institutional implementation procedures were introduced.

Accordingly, this study aimed to examine three distinct domains over the six months following the introduction of a 3D printer with minimal formal implementation support in a rehabilitation hospital: (1) occupational therapists' perceptions of 3D printers, (2) perceived barriers and enabling conditions related to implementation, and (3) early usage patterns.

## Materials and methods

### Study design

This single-site pre–post observational study compared occupational therapists' perceptions and perceived barriers and enabling conditions associated with 3D printer introduction in a rehabilitation hospital. The study focused on the early implementation phase following the introduction of the printer with minimal formal implementation support. This support included brief basic operational instruction for one representative occupational therapist and access to pre-existing design files, but did not include structured staff training, CAD instruction, formal usage recommendations, workflow integration, or dedicated institutional support.

### Participants

Participants were 54 occupational therapists (15 men, 39 women) working at a 551-bed rehabilitation hospital, which included a 200-bed recovery-phase rehabilitation ward. All participants belonged to the occupational therapy department but worked across different clinical areas within the occupational therapy service. The sample size was determined by the number of eligible occupational therapists at the study site, and no *a priori* power-based sample-size calculation was performed. Clinical experience was assessed using a Likert-type scale; most had ≤5 years. All participants provided written informed consent at baseline. A questionnaire survey was conducted before and 6 months after installation; only participants who completed both surveys were included in the analysis.

### 3D printer installation and implementation procedure

In approximately July 2024, a Bambu Lab X1-Carbon 3D printer (Bambu Lab, Shenzhen, Guangdong, China) was installed in a shared, easily accessible space in the occupational therapy department. The printer was available for use by all occupational therapists in the department. No formal booking system, prior authorization procedure, or mandatory supervision was required for printer use. Staff were permitted to use the 3D printer during paid working hours, although no dedicated time was allocated specifically for 3D-printing activities. PLA and ABS filaments were available during the study period. Material costs were covered by the hospital. Routine maintenance and first-line troubleshooting were handled by the designated staff member serving as the point of contact for printer use; when issues could not be resolved, the researcher provided additional support. In addition to preloaded manufacturer models, STL files from the “Assistive Devices” category (15) on the FabLab Shinagawa official website were transferred to the printer via microSD card. This configuration enabled occupational therapists to output existing designs without using CAD software. No design tasks requiring dimensional or shape modification were included, and no formal instruction in CAD-based design or patient-specific modification was provided. The available files therefore supported basic trial printing, but not independent design or customization.

### Implementation conditions and support

At the time of installation, brief instruction in basic printer operation—covering startup, model selection, printing, and filament replacement—was provided to one representative occupational therapist. This occupational therapist was included in the paired analysis. No instruction in CAD data creation, advanced settings, or troubleshooting was given. Operational procedures were shared informally among staff through verbal communication during routine work, without formal manuals, training sessions, or workshops.

Use of the 3D printer was at the discretion of individual occupational therapists; no goals or recommendations regarding frequency or purpose of use were defined. No institutional measures were introduced, such as assigning dedicated personnel, guaranteeing access during working hours, or linking outputs to medical reimbursement. Participants were not provided with formal information or instruction regarding medical reimbursement or relevant disability-welfare benefit schemes for 3D-printed assistive devices. No fees were charged to patients for the 3D-printed items produced during the study period. Accordingly, the implementation condition was characterized as printer availability with minimal formal support rather than an intervention-free condition.

### Assessment

Responses were rated on a 5-point Likert scale (1 = strongly agree; 5 = strongly disagree). Assessments were conducted at baseline and approximately six months after installation, with the follow-up questionnaire administered in January 2025, using the same self-administered questionnaire. The study-specific questionnaire included items concerning perceptions of 3D printers, self-rated skills, perceived challenges, enabling conditions, and perceived advantages and disadvantages. For questionnaire domains concerning perceived challenges, enabling conditions, advantages, and disadvantages, participants were instructed to select up to three items that they considered most applicable. The questionnaire format limited responses to a maximum of three items; however, a formal methodological rationale for selecting this maximum was not documented during questionnaire development. Participants were not required to select exactly three items. Consequently, non-selection did not necessarily indicate disagreement or the absence of a particular perception. Because the number of selectable responses was constrained, selections within each question set were not independent; selection of one item could affect whether another item was selected. The questionnaire was developed for exploratory use in this study by a multidisciplinary team with medical and engineering expertise. It was originally developed and administered in Japanese. No separate formal expert review, content-validity assessment, pilot testing, psychometric validation, or reliability testing was conducted. Because the questionnaire was administered in its original Japanese version, no translation or back-translation procedure was required or performed. A formal item-by-item rationale for the wording of each questionnaire item was not documented during questionnaire development. It was therefore not considered a validated measure of acceptability, feasibility, appropriateness, or adoption. No single implementation or technology-acceptance framework prospectively guided questionnaire development or outcome selection. The complete set of questionnaire items is provided in the Supplementary Material.

### Assessment of actual usage

A comprehensive usage log system was not implemented; therefore, the total number of print jobs, frequency and timing of use, number of individual users, and number of outputs provided to patients could not be accurately determined. After the six-month observation period, usage was assessed by reviewing the printer's retrievable output history and available printed items. The available records and printed items were reviewed and descriptively classified by one researcher; independent duplicate classification was not performed. The review was used only to describe verifiable output types and general patterns of early use. These records were not treated as a quantitative measure of individual adoption, frequency of use, or routine clinical implementation. Because individual-level printer usage was not recorded, it was not possible to determine whether the occupational therapist who received the initial operational instruction used the printer more frequently than other participants.

### Ethical considerations

This study was approved by the University Ethics Review Committee (approval number: 2023-61). Participants were informed of the study purpose, voluntary participation, right to withdraw, and handling of personal information. Written informed consent was obtained, and all data were anonymized in accordance with the Declaration of Helsinki.

### Statistical analyses

Analyses were conducted using a complete-case approach and included only participants with both baseline and follow-up data. Normality was assessed using the Shapiro–Wilk test. Paired Likert-scale responses were analyzed using the Wilcoxon signed-rank test, and paired binary responses to multiple-response questionnaire items were analyzed using the McNemar test. The Holm method was used to adjust *p*-values for multiple comparisons within defined questionnaire families: across all 12 items in the perceived-challenges domain, across all 12 items in the enabling-conditions domain, and separately across the six advantages items and the six disadvantages items. These multiplicity families were defined during the revision process and had not been prespecified before the original analysis. Accordingly, the multiplicity-adjusted item-level analyses were considered exploratory. Because participants could select up to three responses within each multiple-response question set, non-selection of an item was not interpreted as disagreement. Responses exceeding the instructed maximum of three selections were treated as invalid for the affected question set. For paired analyses, the corresponding participant was excluded from both assessments for that question set. Because the number of selectable responses was constrained, selections within each question set were not independent; selection of one item could affect whether another item was selected. Statistical analyses were performed using SPSS version 29 (IBM Corp., Armonk, NY, USA), and a two-sided significance level of 0.05 was applied.

### Writing assistance disclosure

An artificial intelligence-based language model (ChatGPT-5.2, OpenAI) was used to assist with grammar checking and language refinement. All content was reviewed and approved by the authors, who take full responsibility for the manuscript.

## Results

### Participant flow and follow-up

Forty-three (79.6%) participants completed the 6-month follow-up [12 [27.9%] men, 31 [72.1%] women], and paired data from these participants were analyzed. Eleven participants (20.4%) did not complete the follow-up. The main recorded reasons for loss to follow-up were leave of absence and resignation; however, the exact number of participants corresponding to each reason was not documented. Whether any participant explicitly declined the follow-up assessment was not separately recorded. The participant flow is shown in Figure 1. Table 1 presents the participants' characteristics. Most participants had ≤5 years of clinical experience (22 participants, 51.2%) and were working in recovery-phase rehabilitation (35 participants, 81.4%).

**Figure 1:**
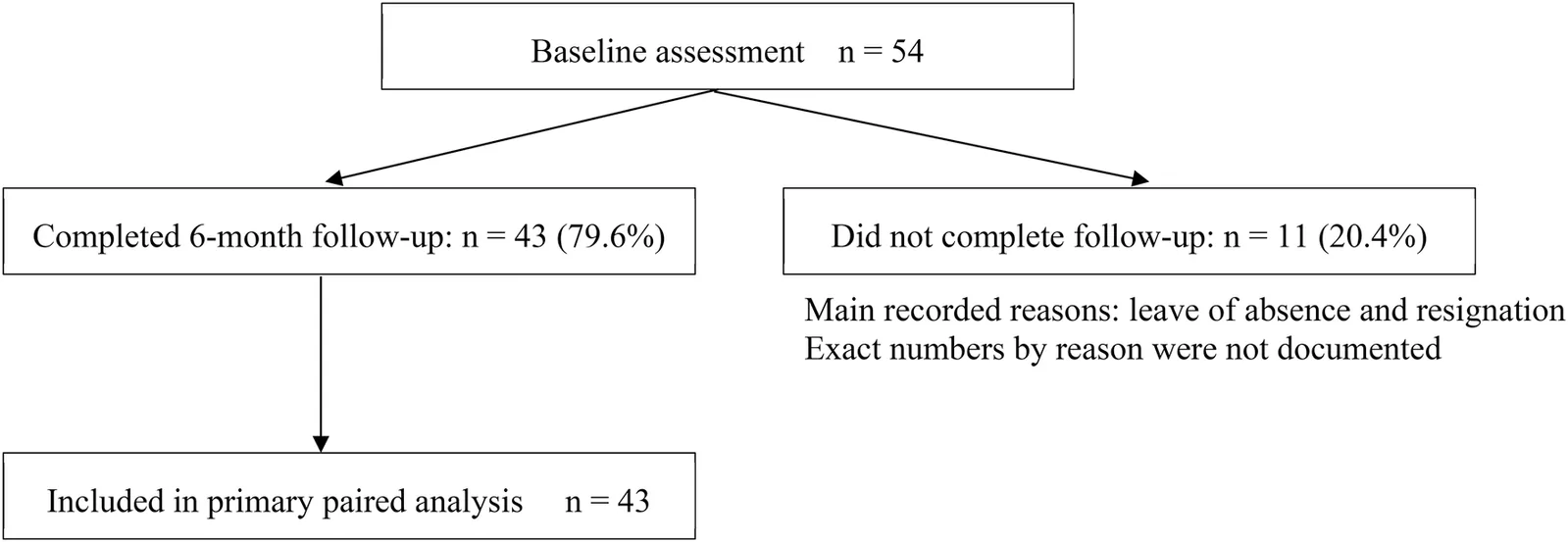
Participant flow through the study.

**Table 1:** Background characteristics of participants.

Question Item	Classification	n	%
Sex	Male	12	27.9
	Female	31	72.1
How many years of experience do you have as an occupational therapist?	≤5 years	22	51.2
	6–10 years	10	23.3
	11–15 years	3	7.0
	16–20 years	6	14.0
	21–25 years	2	4.7
Disease stage of patients treated at the respondent's facility	Preventive phase	0	0
	Acute phase	0	0
	Recovery phase	35	81.4
	Community-based (long-term) phase	5	11.6
	End-of-life phase	1	2.3
	Other	2	4.7
Do you like making things, such as crafting?	Strongly agree	9	20.9
	Agree	23	53.5
	Neutral	6	14.0
	Disagree	5	11.6
	Strongly disagree	0	0
Do you think you are good at making things?	Strongly agree	1	2.3
	Agree	13	30.2
	Neutral	18	41.9
	Disagree	11	25.6
	Strongly disagree	0	0
Do you like working with computers and other electronic devices?	Strongly agree	2	4.7
	Agree	13	30.2
	Neutral	13	30.2
	Disagree	13	30.2
	Strongly disagree	2	4.7
Experience utilizing assistive devices and splints	Not much	16	37.2
	A few times a year	12	27.9
	About once a month	14	32.6
	About once a week	0	0
	Several times a week	1	2.3
How often do you provide assistive devices and splints to your patients (including purchasing, making, and adjusting them)?	Not much	16	37.2
	A few times a year	17	39.5
	About once a month	8	18.6
	About once a week	2	4.7
	Several times a week	0	0
Please tell us the main target diseases when providing self-help devices and splints. ＊Multiple responses allowed	Stroke	32	74.4
	Diseases and traumas of the musculoskeletal system	11	25.6
	Spinal cord injury	24	55.8
	Neuromuscular disease	7	16.3
	Amputation (trauma, hematologic injury)	0	0
	Pediatric diseases	0	0
	Rheumatoid arthritis	3	7.0
	Cardiovascular disease	1	2.3
	Respiratory disease	1	2.3
	Feeding and swallowing disorders	0	0
	Cancer (malignant tumor)	0	0
	Internal disorder	1	2.3
	Developmental disorders	0	0
	Mental disorders	0	0
	Dementia and other geriatric disorders	1	2.3

(*n* = 43).

Individual characteristics related to familiarity with 3D printers were also examined. At baseline, two participants (4.7%) reported previous experience using a 3D printer, compared with three participants (7.0%) at six months. Because the questionnaire did not identify whether participants had used the installed printer, these responses were not interpreted as a measure of individual printer use. No participants owned a 3D printer. At baseline, 13 participants (30.2%) reported intentionally gathering information about 3D printers, compared with 20 participants (46.5%) at six months. This descriptive difference was not interpreted as evidence of a pre–post change. Baseline comparisons between participants who completed the six-month follow-up and those who did not showed no statistically significant differences in sex distribution (Fisher's exact test, *p* = 1.000), clinical experience (Mann–Whitney *U* = 188.5, *p* = 0.273), perceived difficulty of using a 3D printer (*U* = 180.5, *p* = 0.188), perceived usefulness in occupational therapy practice (*U* = 201.5, *p* = 0.377), or future intention to use a 3D printer (*U* = 210.5, *p* = 0.521).

### Usage patterns of the 3D printer

Among the verifiable outputs, the identified items consisted of publicly available STL files or default printer models. No use involving the creation of new CAD data or original shape design was observed. When classified by function, outputs included assistive devices and aids for activities of daily living, such as bottle and can openers; spoon, bottle, and toothbrush holders; shoe horns; zipper lock aids; hand soap aids; and easy-to-use zippers. Mobility- and travel-related items included walker pockets and shopping bag handles. Tools for object holding and manipulation included card holders and writing guides. Exercise cones and figures were produced for training, prototyping, and demonstration purposes.

The verifiable outputs were primarily simple items intended as alternatives or supplements to commercially available products. No shape modifications or dimensional adjustments tailored to individual patient requirements were performed. Among the verifiable outputs, patient-facing use appeared limited, and the available items consisted mainly of prototypes produced for test printing or usability confirmation. Because comprehensive usage records were unavailable, the total number of outputs provided to patients could not be determined. Overall, 3D printer use was limited to exploratory and trial use of existing design data, without progression to routine clinical application.

### Shift in perceptions regarding 3D printers

Changes in perceptions regarding 3D printers are presented in Table 2. At six months post-installation, participants showed significantly less agreement with the statement “Do you think using a 3D printer is difficult?” (mean score, 2.26–2.63; adjusted *p* = 0.047). Agreement with the statement that a 3D printer could be useful in occupational therapy practice also decreased significantly (mean score, 1.74–2.00; adjusted *p* = 0.044). No statistically significant change was observed in intention to use a 3D printer in future occupational therapy practice (mean score, 1.95–2.16; adjusted *p* = 0.063).

**Table 2:** Changes in occupational therapists' perceptions (Pre–post).

Question Item	Pretest	Posttest	Mean Change	*p* ^a^	Effect size（r）
	Mean ± SD	Mean ± SD	(95％ CI)		
Do you think using a 3D printer is difficult?	2.26 ± 0.69	2.63 ± 0.79	0.37 (0.08–0.67)	**0**.**047**	0.369
Do you think a 3D printer can be useful in your occupational therapy practice?	1.74 ± 0.58	2.00 ± 0.62	0.26 (0.04–0.47)	**0**.**044**	0.350
Do you want to use (continue using) a 3D printer in occupational therapy in the future?	1.95 ± 0.62	2.16 ± 0.65	0.21 (−0.01–0.43)	0.063	0.283

(*n* = 43). Scale: 1 = strongly agree, 2 = agree, 3 = neutral, 4 = disagree, 5 = strongly disagree.

a *P*-values were calculated using the Wilcoxon signed-rank test and adjusted using the Holm method. Effect size was calculated as r = |Z|/√N.

### Changes in perceived barriers, enabling conditions, advantages, and disadvantages

(1) Perceived Challenges Related to Implementation (Table 3)

**Table 3:** Changes in perceived challenges related to 3D printer implementation.

What are some of the challenges in implementing 3D printers within occupational therapy?	Number selected		Not selected → Selected	Selected → Not selected	Paired OR (Exact 95% CI)	*p* ^a^
Question Item	Pretest	Posttest				
(A) The environment or system: Please select up to 3 items that apply more to you						
There is no 3D printer in the workplace	38	9	0	29	0.00 (0.00–0.14)	**<0**.001*
There is no designated place for a 3D printer in the workplace	1	2	1	0	∞ (0.03–∞)	1.000
There is no budget to purchase a 3D printer for the workplace	9	7	6	8	0.75 (0.21–2.46)	1.000
There is no time flexibility during office hours to use the 3D printer to create products	13	34	22	1	22.00 (3.56–907.95)	**<0**.001*
There is no medical reimbursement for products created using a 3D printer	4	17	16	3	5.33 (1.53–28.56)	**0**.044*
There is no budget to purchase materials for the 3D printer	8	7	6	6	1.00 (0.27–3.74)	1.000
Mean number of items selected per participant	1.70	1.77	-	-	-	-
(B) The knowledge and skills: Please select up to 3 items that apply more to you						
I don't know how to use a 3D printer	38	30	2	10	0.20 (0.02–0.94)	0.347
Can't visualize what can be created with a 3D printer	12	13	7	6	1.17 (0.34–4.20)	1.000
Can't generate 3D data for objects that can be produced with a 3D printer	28	25	5	8	0.63 (0.16–2.17)	1.000
I don't understand the specific processes involved in using a 3D printer in occupational therapy	20	17	6	9	0.67 (0.20–2.10)	1.000
I don't know how objects made with a 3D printer can be applied in occupational therapy	1	5	5	1	5.00 (0.56–236.49)	1.000
Unaware of any examples of 3D printer utilization in occupational therapy	7	13	10	4	2.50 (0.72–10.92)	1.000
Mean number of items selected per participant	2.47	2.40	-	-	-	-

(*n* = 43).

a *p*-values were calculated using McNemar's test and adjusted using the Holm method across all 12 items within the perceived-challenges domain. Paired odds ratios were calculated from discordant pairs as the number changing from not selected to selected divided by the number changing from selected to not selected; exact 95% confidence intervals are reported. The mean number of items selected per participant is reported separately for each multiple-response subdomain.

Among the 12 items assessing perceived challenges related to 3D printer implementation, significant changes from baseline to six months were observed in three items. The number of participants selecting “No 3D printer in the workplace” decreased from 38 (88.4%) to 9 (20.9%) (adjusted *p* < 0.001). The number selecting “Insufficient time during clinical hours to create products using a 3D printer” increased from 13 (30.2%) to 34 (79.1%) (adjusted *p* < 0.001). Selection of “No medical reimbursement for products created using a 3D printer” increased from 4 (9.3%) to 17 (39.5%) (adjusted *p* = 0.044). No significant changes were observed in the remaining items, including lack of workspace, insufficient purchase funds, material costs, and lack of knowledge regarding printer operation (all adjusted *p* ≥ 0.05). Because participants could select up to three items, these results indicate changes in item selection or prioritization and should not be interpreted as direct measures of agreement or disagreement.

(2) Perceived Conditions That May Facilitate Clinical Use (Table 4).

**Table 4:** Changes in perceived conditions that May facilitate clinical 3D-printer Use.

Under what conditions do you think a 3D printer could be introduced?	Number selected		Not selected → Selected	Selected → Not selected	Paired OR (Exact 95% CI)	*p* ^a^
Question Item	Pretest	Posttest				
(A) Environment maintenance: Please select up to 3 items that apply more to you						
A 3D printer is available nearby	32	16	1	17	0.06 (0.001–0.38)	**0**.002*
Occupational therapists are permitted to use 3D printers during working hours as part of their occupational therapy duties	17	25	11	3	3.67 (0.97–20.47)	0.574
The products created by the 3D printers are associated with reimbursement for medical treatment	2	17	17	2	8.50 (2.02–75.85)	**0**.008*
There is sufficient time available to learn about 3D printers	25	23	6	8	0.75 (0.21–2.46)	1.000
There is someone nearby who can provide instruction on 3D printers	31	32	9	8	1.13 (0.39–3.35)	1.000
Can claim the cost of the 3D printer for the workplace or clients	9	7	4	6	0.67 (0.14–2.81)	1.000
Mean number of items selected per participant	2.70	2.79	-	-	-	-
(B) Acquisition of knowledge and skills: Please select up to 3 items that apply more to you						
There are training sessions and videos (e.g., on YouTube) covering the basic knowledge of 3D printers	27	20	7	14	0.50 (0.17–1.32)	1.000
There are practical training sessions on the operation and utilization of 3D printers	32	30	8	10	0.80 (0.27–2.25)	1.000
There are workshops and training videos (e.g., on YouTube) focusing on the application of 3D printers in occupational therapy	8	6	4	6	0.67 (0.14–2.81)	1.000
There are practical training sessions on designing self-help devices independently (using CAD software)	20	25	15	10	1.50 (0.63–3.73)	1.000
There are opportunities and resources to explore numerous case studies demonstrating the application of 3D printers in occupational therapy	7	15	12	4	3.00 (0.91–12.76)	0.691
There are opportunities and resources to view numerous actual products and photos of self-help devices created with a 3D printer in occupational therapy	14	12	7	9	0.78 (0.25–2.35)	1.000
Mean number of items selected per participant	2.51	2.51	-	-	-	-

(*n* = 43).

a *p*-values were calculated using McNemar's test and adjusted using the Holm method across all 12 items within the perceived enabling-conditions domain. Paired odds ratios were calculated from discordant pairs as the number changing from not selected to selected divided by the number changing from selected to not selected; exact 95% confidence intervals are reported. The mean number of items selected per participant is reported separately for each multiple-response subdomain.

Among the 12 items concerning conditions that participants considered potentially helpful for clinical 3D-printer use, significant changes from baseline to six months were observed in two items. The number of participants selecting “Having a 3D printer readily available” decreased from 32 (74.4%) to 16 (37.2%) (adjusted *p* = 0.002). The number selecting “the produced work is linked to medical reimbursement” increased from 2 (4.7%) to 17 (39.5%) (adjusted *p* = 0.008). No significant changes were observed in the remaining items, including having sufficient time to learn 3D printing, having an instructor readily available, and being able to claim expenses (all adjusted *p* ≥ 0.05). As participants could select up to three options, these item-level changes may partly reflect shifts in prioritization among the available conditions.

(3) Perceived Advantages and Disadvantages (Table 5).

**Table 5:** Perceived advantages and disadvantages of 3D printers (pre–post).

Question Item	Number selected		Not selected → Selected	Selected → Not selected	Paired OR (Exact 95% CI)	*p* ^a^
	Pretest	Posttest				
What do you think the advantages of using a 3D printer (Choose up to 3 items)?						
Unlike ready-made products, it can be tailored to the individual needs and condition of the patient	36	31	3	8	0.38 (0.06–1.56)	0.898
Once a product is created, it can be duplicated as many times as necessary	23	17	5	11	0.45 (0.12–1.42)	0.898
Dimensions and angles can be fine-tuned during the manufacturing process	21	10	3	14	0.21 (0.04–0.77)	0.076
The appearance of the product can be enhanced for aesthetics	16	11	2	7	0.29 (0.03–1.50)	0.898
Uniform production regardless of skill level	19	23	13	9	1.44 (0.57–3.83)	0.898
In some cases, data can be downloaded and used without the need to start from scratch	11	16	9	4	2.25 (0.63–10.00)	0.898
Mean number of items selected per participant	3.00	2.57	-	-	-	-
What do you think are the disadvantages of using a 3D printer (Choose up to 3 items)?						
It takes time to create CAD data	31	21	2	12	0.17 (0.02–0.75)	0.078
Creating CAD data can be challenging	33	27	6	12	0.50 (0.15–1.44)	0.952
It takes time to adjust the 3D printer	15	12	6	9	0.67 (0.20–2.10)	1.000
Adjusting the 3D printer can be difficult	21	12	5	14	0.36 (0.10–1.05)	0.318
Printing takes time	8	9	9	8	1.13 (0.39–3.35)	1.000
It is costly for materials and maintenance	15	14	7	8	0.88 (0.27–2.76)	1.000
Mean number of items selected per participant	2.93	2.26	-	-	-	-

(*n* = 42).

a *p*-values were calculated using McNemar's test and adjusted using the Holm method separately within the advantages and disadvantages question sets. Paired odds ratios were calculated from discordant pairs as the number changing from not selected to selected divided by the number changing from selected to not selected; exact 95% confidence intervals are reported. The mean number of items selected per participant is reported separately for each question set. One participant in the advantages question set and a different participant in the disadvantages question set selected four items at posttest, exceeding the instructed maximum of three selections. The affected participant was excluded from the paired analysis for that question set.

No statistically significant changes were observed in the selection of perceived advantages or disadvantages of 3D printers (all adjusted *p* ≥ 0.076). Because participants could select up to three options, item-level numerical changes may reflect shifts in prioritization and were interpreted as exploratory.

## Discussion

This study explored early usage patterns and changes in occupational therapists' perceptions following the introduction of a 3D printer with minimal formal implementation support. It did not evaluate a comprehensive implementation strategy aimed at establishing routine clinical use.

After installation, the perceived barrier that no 3D printer was available in the workplace decreased markedly. In contrast, institutional and operational challenges, including insufficient time during clinical work and the absence of medical reimbursement for 3D-printed products, became more prominent. Participants showed less agreement that using a 3D printer was difficult, while agreement that 3D printing was useful in occupational therapy practice also decreased. These findings occurred alongside limited exploratory use and may reflect an early implementation stage in which basic familiarity increased without corresponding progression toward routine clinical application.

### Relationship between actual usage and changes in perception during the early implementation phase

Actual use of the 3D printer primarily involved printing with publicly available STL files or pre-installed models; no new CAD designs or individual shape modifications were applied. Most outputs consisted of assistive devices and aids supporting activities of daily living, such as bottle openers and spoon holders. Few items were provided to patients, and use remained largely for prototyping or exploration.

This usage pattern reflects a typical early stage preceding routine clinical application, in which engagement is limited to exploratory use of existing design data. Initial use of 3D printers in occupational therapy often begins with simple fabrication of assistive devices based on existing data, prior to more advanced or individualized applications (6, 8), as identified in the present study.

The reduced agreement that using a 3D printer was difficult may indicate that exposure to basic printer operation lowered initial uncertainty or psychological barriers. However, operational competence was not directly assessed, and this finding should not be interpreted as evidence that participants acquired practical proficiency. The limited use observed in this study involved mainly pre-existing design files and simple printing procedures. Thus, familiarity with basic operation may have increased even though participants did not progress to more advanced activities such as CAD-based design or patient-specific customization. Previous educational studies have suggested that structured instruction can improve understanding, self-efficacy, and engagement with 3D-printing technology (7–9), indicating that hands-on exposure alone and formal skill development should be considered distinct stages of implementation.

Because comprehensive usage logs were not collected, the total number of print jobs, frequency of use, number of individual users, and proportion of outputs provided to patients could not be determined accurately. The available printer history and stored outputs indicated that use was mainly exploratory and involved pre-existing designs, but these records should not be interpreted as a quantitative measure of individual adoption or routine clinical use. They may have omitted deleted or overwritten records while also including test prints, repeated prototypes, or unsuccessful attempts. Therefore, the observed outputs were used only to describe the types of early use that could be verified.

### Declining utility assessment and emergence of institutional challenges

Agreement that 3D printing was useful in occupational therapy practice decreased after installation. One possible explanation is that participants had limited opportunities to experience the clinical benefits most commonly associated with 3D printing. The outputs identified during the observation period were predominantly simple items produced from existing design files and used as alternatives or supplements to commercially available products. The potential advantages of 3D printing, including patient-specific customization, precise dimensional adjustment, and iterative design, were therefore not fully experienced. This finding may reflect a gap between initial expectations and actual early use rather than a rejection of the technology itself. However, because perceived usefulness was assessed using a single non-validated item, this interpretation should remain cautious. Although the changes in perceived difficulty and usefulness were statistically significant, their absolute magnitudes were small (0.37 and 0.26 points, respectively, on the five-point scale). The practical or clinical importance of changes of this magnitude is uncertain and should not be inferred from statistical significance alone. In addition, future intention to use a 3D printer did not change significantly (adjusted *p* = 0.063), and this finding was not interpreted as evidence of a trend.

Ventola (2) noted that the broader implementation of 3D printers in medical settings requires attention to time, cost, institutional approval, and technological utility. In the present study, insufficient time during clinical work and perceived lack of medical reimbursement were selected more frequently after installation. In Japan, reimbursement for assistive devices is not determined simply by whether an item is produced using 3D printing. Certain assistive devices may be supported through separate disability-welfare schemes, such as the prosthetic and orthotic device benefit system or municipal daily-living equipment programs, depending on the type of device, eligibility criteria, and local procedures. In the present study, no fees were charged to patients for the printed items, and the study did not evaluate whether the costs of such low-cost assistive devices would otherwise be borne by the institution, the patient, or a public benefit scheme. These findings suggest that institutional and operational issues became more salient once participants had direct exposure to the printer. However, because the study did not directly assess usability, workflow integration, or participants' knowledge of reimbursement rules, the questionnaire responses should be interpreted as perceptions rather than evidence that reimbursement was objectively unavailable. Accordingly, the mechanisms underlying these changes cannot be determined.

Selection of the item “A 3D printer is available nearby” decreased after installation, despite the printer being physically present in the department. This apparently paradoxical result may reflect ambiguity in the item wording, changes in participants' prioritization among the limited response options, or a distinction between physical proximity and practical accessibility. Because these possibilities were not directly examined, the finding should not be interpreted as evidence of subjective availability. Rather, it highlights a limitation in how this item functioned and should be interpreted cautiously.

### Significance of introduction with minimal formal support

A key characteristic of this study was that the 3D printer was introduced with minimal formal implementation support. One occupational therapist received brief instruction in basic operation, existing design files were made available, and operational information was subsequently shared informally among staff. However, no structured training, CAD instruction, formal usage recommendations, dedicated support personnel, workflow integration, or institutional implementation procedures were provided. The study therefore did not examine an intervention-free condition, but rather an early introduction phase with limited formal support.

A nationwide survey in Japan reported that workplace availability of 3D printers was strongly associated with occupational therapists' previous experience of using them (10). However, equipment availability represents only one component of implementation. In the present study, changes in perceptions and observed usage patterns were examined following the introduction of a printer under limited-support conditions. Because this was an uncontrolled single-group pre–post study, the observed changes cannot be attributed specifically to printer availability, and may also have been influenced by informal peer communication, exposure to preloaded design files, participation in the study, external information, or the passage of time.

Within these limitations, the findings indicate that making a 3D printer available with minimal formal support was not accompanied by clear progression toward routine clinical use during the six-month observation period. The results do not establish which additional implementation components are necessary or sufficient. Nevertheless, they support further investigation of structured education, technical assistance, workflow integration, dedicated work time, and institutional arrangements as potential components of future implementation strategies.

### Challenges and future prospects for clinical implementation

This study found that the available 3D printer was used primarily for prototyping simple assistive devices based on existing design data during the six-month observation period. Future implementation studies should evaluate educational approaches that support practical skill acquisition, including CAD-based design, as well as peer-based mechanisms for sharing experiences and examples of use. In the context of prosthetic and orthotic fabrication using 3D printing, clinical implementation may depend not only on technical feasibility but also on cost considerations and practical implementation factors (16). Recent work has also demonstrated the feasibility of low-cost additive manufacturing for assistive medical devices, further highlighting the potential importance of fabrication cost and local accessibility in broader clinical implementation (17). The institutional and operational issues reported more frequently after installation in the present study are consistent with this perspective. However, the present findings do not establish that any specific educational or institutional strategy would be sufficient to promote routine clinical use. Progression toward routine patient-facing use would also require consideration of implementation issues beyond education, time, and reimbursement. These include material safety, hygiene and cleaning procedures, product durability, quality assurance, patient-specific risk assessment, responsibility for device failure, data governance for patient-specific designs, regulatory classification, institutional approval, maintenance and calibration of equipment, and intellectual property or licensing requirements for downloaded design files. Hospital-based and point-of-care 3D-printing literature has similarly emphasized the importance of quality-management systems, patient safety, process validation, documentation, and appropriate regulatory oversight (18, 19). These issues were not systematically evaluated in the present study and should be addressed in future clinical implementation research.

### Study significance and limitations

This study examined the early clinical introduction of a 3D printer in a rehabilitation hospital under conditions of minimal formal implementation support. While previous studies have often examined 3D-printing use in conjunction with educational programs and active promotion, empirical research describing early use under limited-support conditions remains limited. Accordingly, this study described usage patterns and perceptual changes during a six-month period in which brief basic instruction and pre-existing design files were available, but no structured training, workflow integration, dedicated personnel, or formal institutional implementation procedures were provided.

Alongside the questionnaire assessment, usage patterns were described using retrievable printer records and available printed items. The available records indicated mainly exploratory use of pre-existing designs. Although these observations occurred alongside changes in selected perceptions, the study design does not establish a direct relationship between perceptual change and printer use.

Furthermore, by examining early experiences after a printer became available in the workplace—a major barrier identified in a prior nationwide survey—this study provides exploratory information about occupational therapists' perceptions and trial use under limited-support conditions. The findings may help inform the design of future studies evaluating educational, technical, workflow, and institutional support for clinical 3D-printing use.

However, this study had several limitations. First, it was conducted at a single rehabilitation hospital with a relatively small sample, and caution is required when generalizing the findings, as usage patterns and perceptions may vary by institutional size, organizational culture, operational structure, staffing, and access to technical support. The sample was determined by the number of eligible occupational therapists at the site and may have been insufficient to detect small changes or support subgroup analyses. Accordingly, non-significant findings should not be interpreted as evidence that no meaningful change occurred, and the results should be regarded as site-specific exploratory observations. Second, the study used an uncontrolled single-group pre–post design. Therefore, changes observed over six months cannot be attributed specifically to printer introduction and may have been influenced by participation in the study, informal communication among staff, exposure to the available design files, external information, or the passage of time. The observed pre–post differences should therefore be interpreted as temporal associations rather than causal effects of introducing the printer. Third, because comprehensive user-recorded logs were not collected, quantitative evaluation of print volume, frequency of use, or individual user activity was not possible. The available printer history also could not distinguish successful clinical outputs from test prints, repeated prototypes, failed attempts, or deleted records. Consequently, the study could describe only verifiable output types and could not measure individual adoption, sustained use, or routine clinical implementation. Accordingly, descriptions of limited or exploratory use should not be interpreted as quantitative estimates of usage intensity or adoption. Regulatory, safety, and liability requirements associated with patient-facing use of 3D-printed assistive devices were not systematically evaluated in this study and may vary according to the intended use, device characteristics, and applicable institutional or regulatory requirements. Other implementation considerations relevant to routine patient-facing use, including material safety, hygiene and cleaning procedures, product durability, quality assurance, patient-specific risk assessment, responsibility for device failure, data governance for patient-specific designs, institutional approval, equipment maintenance and calibration, and intellectual property or licensing requirements for downloaded design files, were also not systematically evaluated. Fourth, participant characteristics that may influence technology use—including age, digital literacy, computer competence, previous CAD experience, technology anxiety, workload, employment status, employment role, and individual exposure to the trained representative—were not comprehensively collected. Unmeasured differences in these characteristics may have affected both perceptions and use. Therefore, the study cannot determine whether the observed responses differed according to individual technological experience, workload, or other participant-level factors. Fifth, the questionnaire had not undergone formal validity or reliability testing, limiting confidence in the measured perceptual changes. The items should therefore be regarded as exploratory and cannot be assumed to represent validated measures of acceptability, feasibility, appropriateness, or adoption. Ambiguity in item wording may also have contributed to apparently paradoxical findings, such as the reduced selection of printer availability after installation. Consequently, small changes in questionnaire responses should be interpreted cautiously and should not be treated as precise estimates of established implementation constructs. Sixth, participants could select up to three options in the domains concerning challenges, enabling conditions, advantages, and disadvantages. Changes in the selection of individual items may therefore reflect shifts in prioritization among available responses rather than genuine increases or decreases in agreement. Item-level McNemar analyses should consequently be interpreted as exploratory. Seventh, 11 of the 54 baseline participants did not complete the follow-up survey. Although leave of absence and resignation were the main recorded reasons, no statistically significant baseline differences were identified between completers and non-completers in sex, clinical experience, perceived difficulty, perceived usefulness, or future intention to use a 3D printer. Nevertheless, the possibility of attrition bias cannot be excluded because these comparisons were limited to available baseline variables and complete-case analysis was used. In addition, information linking specific reasons for loss to follow-up with participants' clinical experience or workplace role was not retained in sufficient detail to permit a reliable reason-specific analysis. Therefore, the follow-up findings may not fully represent the experiences or perceptions of all baseline participants. Eighth, the six-month observation period may have been insufficient to capture long-term changes in skills, workflow integration, continued use, or institutional implementation. Accordingly, the absence of clear progression toward routine clinical use during this period should not be interpreted as evidence that such progression would not occur with longer observation. Finally, the implementation condition involved minimal formal support rather than physical access alone, because one occupational therapist received basic operational instruction, existing design files were provided, and information was shared informally. The findings should therefore be interpreted as descriptive observations following introduction under limited-support conditions, rather than evidence of the isolated or causal effect of making the printer available in the workplace.

## Conclusion

This single-site pre–post observational study found that the introduction of a 3D printer with minimal formal implementation support was accompanied by selected changes in occupational therapists' perceptions and perceived implementation-related conditions, while observed use remained exploratory and relied primarily on existing design files. No clear progression toward routine clinical use was identified during the six-month observation period.

These findings provide exploratory information for future studies evaluating whether structured educational, technical, workflow, and institutional support can facilitate more sustained clinical use. Such studies should use validated implementation measures and quantitative usage records to assess these strategies prospectively.

## Data Availability

The datasets presented in this article are not readily available due to ethical restrictions. Although direct identifiers are not included, the data were collected from a single institution with a relatively small sample size, which may pose a risk of re-identification. Secondary use of the data requires additional approval from the ethics review board. Requests to access the datasets should be directed to the corresponding author.
