# Supplementary material for: Early occupational therapy use of a 3D printer introduced with minimal formal support: a single-site pre–post observational study
Source: Front Rehabil Sci. 2026 Sep 22;7:1859684. doi: 10.3389/fresc.2026.1859684 (PMC13638619; doi:10.3389/fresc.2026.1859684)
Supplement: Supplementary file 1 [file Supplementaryfile1.docx]

**Supplementary Material 1**

The questionnaire items

Q1）Have you used a 3D printer before?

①Yes, ②No

Q2）Do you own a 3D printer?

①Yes/Held at the workplace
　②Yes/Privately held
　③Yes/Held both at work and privately
　④No/Not held

Q3）Have you intentionally gathered information about 3D printers, such as watching videos, visiting websites, or attending training sessions?

①I've done it often
　②I have done it
　③I've never done it

Q4）How much knowledge do you have about 3D printers in general?　Please answer on a scale of 0 to 10.

0 : I've never seen or heard of it　〜　10 : I know how to use it and an overview of what I can make

Q5）Do you think using a 3D printer is difficult?

①Strongly agree
　②Agree
　③Neutral
　④Disagree
　⑤Strongly disagree

Q6）Do you think a 3D printer can be useful in your occupational therapy practice?

①Strongly agree
　②Agree
　③Neutral
　④Disagree
　⑤Strongly disagree

Q7）Do you want to use (continue using) a 3D printer in occupational therapy in the future?

①Strongly agree
　②Agree
　③Neutral
　④Disagree
　⑤Strongly disagree

Q8）What are some of the challenges in implementing 3D printers within occupational therapy?

A）The environment or system : Please select up to 3 items that apply more to you
　　There is no 3D printer in the workplace
　　There is no designated place for a 3D printer in the workplace
　　There is no budget to purchase a 3D printer for the workplace
　　There is no time flexibility during office hours to use the 3D printer to create products
　　There is no medical reimbursement for products created using a 3D printer
　　There is no budget to purchase materials for the 3D printer

B）The knowledge and skills : Please select up to 3 items that apply more to you
　　I don't know how to use a 3D printer
　　Can't visualize what can be created with a 3D printer
　　Can't generate 3D data for objects that can be produced with a 3D printer
　　I don't understand the specific processes involved in using a 3D printer in occupational therapy
　　I don't know how objects made with a 3D printer can be applied in occupational therapy
　　Unaware of any examples of 3D printer utilization in occupational therapy

Q9）Under what conditions do you think a 3D printer could be introduced?

A）Environment maintenance : Please select up to 3 items that apply more to you
　　A 3D printer is available nearby
　　Occupational therapists are permitted to use 3D printers during working hours as part of their occupational therapy duties
　　The products created by the 3D printers are associated with reimbursement for medical treatment
　　There is sufficient time available to learn about 3D printers
　　There is someone nearby who can provide instruction on 3D printers
　　Can claim the cost of the 3D printer for the workplace or clients

B）Acquisition of knowledge and skills : Please select up to 3 items that apply more to you
　　There are training sessions and videos (e.g., on YouTube) covering the basic knowledge of 3D printers
　　There are practical training sessions on the operation and utilization of 3D printers
　　There are workshops and training videos (e.g., on YouTube) focusing on the application of 3D printers in occupational therapy
　　There are practical training sessions on designing self-help devices independently (using CAD software)
　　There are opportunities and resources to explore numerous case studies demonstrating the application of 3D printers in occupational therapy
　　There are opportunities and resources to view numerous actual products and photos of selfhelp devices created with a 3D printer in occupational therapy

Q10）What do you think the advantages of using a 3D printer (Choose 3 items) ?

Unlike ready-made products, it can be tailored to the condition of the object or the subject
　　Once a product is created, it can be duplicated as many times as necessary
　　Dimensions and angles can be fine-tuned during the manufacturing process
　　The appearance of the product can be enhanced for aesthetics
　　Uniform production regardless of skill level
　　In some cases, data can be downloaded and used without the need to start from scratch

Q11）What do you think are the disadvantages of using a 3D printer (Choose 3 items) ?

It takes time to create CAD data
　　Creating CAD data can be challenging
　　It takes time to adjust the 3D printer
　　Adjusting the 3D printer can be difficult
　　Printing takes time
　　It is costly for materials and maintenance
