# Supplementary material for: Early occupational therapy use of a 3D printer introduced with minimal formal support: a single-site pre–post observational study
Source: Front Rehabil Sci. 2026 Sep 22;7:1859684. doi: 10.3389/fresc.2026.1859684 (PMC13638619; doi:10.3389/fresc.2026.1859684)
Supplement: Supplementary file 2 [file Supplementaryfile2.docx]

**Supplementary Material 2**

Detailed distribution of responses to occupational therapists’ perception items at pretest and posttest

|  | Pretest | | | | | Posttest | | | | | | | |  |
| --- | --- | --- | --- | --- | --- | --- | --- | --- | --- | --- | --- | --- | --- | --- |
| Question Item | 1 | 2 | 3 | 4 | 5 |  | 1 | 2 | 3 | 4 | 5 |  |  |  |
| Do you think using a 3D printer is difficult? | 6 （14.0） | 20 （46.5） | 17 （39.5） | 0 （0） | 0 （0） |  | 3 （7.0） | 14 （32.6） | 23 （53.5） | 2 （4.7） | 1 （2.3） |  |  |  |
| Do you think a 3D printer can be useful in your occupational therapy practice? | 14 （32.6） | 26 （60.5） | 3 （7.0） | 0 （0） | 0 （0） |  | 8 （18.6） | 27 （62.8） | 8 （18.6） | 0 （0） | 0 （0） |  |  |  |
| Do you want to use (continue using) a 3D printer in occupational therapy in the future? | 9 （20.9） | 27 （62.8） | 7 （16.3） | 0 （0） | 0 （0） |  | 6 （14.0） | 24 （55.8） | 13 （30.2） | 0 （0） | 0 （0） |  |  |  |
|  | | | | | | | | | | | | |  | |

(n = 43)　 Scale: 1 = strongly agree, 2 = agree, 3 = neutral, 4 = disagree, 5 = strongly disagree.
